# Histone Lysine Demethylases KDM5B and KDM5C Modulate Genome Activation and Stability in Porcine Embryos

**DOI:** 10.3389/fcell.2020.00151

**Published:** 2020-03-10

**Authors:** Werner Giehl Glanzner, Karina Gutierrez, Vitor Braga Rissi, Mariana Priotto de Macedo, Rosalba Lopez, Luke Currin, Naomi Dicks, Hernan Baldassarre, Luis B. Agellon, Vilceu Bordignon

**Affiliations:** ^1^Department of Animal Science, McGill University, Sainte-Anne-de-Bellevue, QC, Canada; ^2^Laboratory of Biotechnology and Animal Reproduction – BioRep, Federal University of Santa Maria (UFSM), Santa Maria, Brazil; ^3^School of Human Nutrition, McGill University, Sainte-Anne-de-Bellevue, QC, Canada

**Keywords:** embryo development, KDM5B, KDM5C, embryo genome activation, DNA damage

## Abstract

The lysine demethylases KDM5B and KDM5C are highly, but transiently, expressed in porcine embryos around the genome activation stage. Attenuation of KDM5B and KDM5C mRNA hampered embryo development to the blastocyst stage in fertilized, parthenogenetically activated and nuclear transfer embryos. While KDM5B attenuation increased H3K4me2-3 levels on D3 embryos and H3K4me1-2-3 on D5 embryos, KDM5C attenuation increased H3K9me1 on D3 embryos, and H3K9me1 and H3K4me1 on D5 embryos. The relative mRNA abundance of *EIF1AX* and *EIF2A* on D3 embryos, and the proportion of D4 embryos presenting a fluorescent signal for uridine incorporation were severely reduced in both KDM5B- and KDM5C-attenuated compared to control embryos, which indicate a delay in the initiation of the embryo transcriptional activity. Moreover, KDM5B and KDM5C attenuation affected DNA damage response and increased DNA double-strand breaks (DSBs), and decreased development of UV-irradiated embryos. Findings from this study revealed that both KDM5B and KDM5C are important regulators of early development in porcine embryos as their attenuation altered H3K4 and H3K9 methylation patterns, perturbed embryo genome activation, and decreased DNA damage repair capacity.

## Introduction

Epigenetic marks regulate crucial events during oocyte to embryo transition (OET) and early embryo development. These include embryo genome activation (EGA), the first cell lineage specification and differentiation, as well as genome repair and stability (Canovas et al., 2012; Bohrer et al., 2014; Ancelin et al., 2016; Dahl et al., 2016; Wasson et al., 2016; Rissi et al., 2019). An epigenetic network is involved in the modulation of mRNA transcription required for normal embryo development (Tadros and Lipshitz, 2009; Ostrup et al., 2013; Eckersley-Maslin et al., 2018). This includes changes in methylation of DNA and histones, such as methylation of lysine 4 (H3K4me), 9 (H3K9me), and 27 (H3K27me) in the histone H3, which are required for normal embryo transcription and programing cell differentiation (Jullien et al., 2014; Dahl et al., 2016; Liu W. et al., 2016; Liu X. et al., 2016; Zhang et al., 2016; Dumdie et al., 2018; Rissi et al., 2019). Although epigenetic marks can be inherited from the gametes (Siklenka et al., 2015; Teperek et al., 2016), how the epigenetic network is regulated to coordinate the different vital functions in early developing embryos remains not well understood.

Studies in embryos produced by somatic cell nuclear transfer (SCNT) revealed that insufficient reprogramming or persistent H3K9 and H3K27 methylation levels are associated with decreased transcriptional activity and impaired embryo development (Matoba et al., 2014; Chung et al., 2015; Xie et al., 2016). There is also evidence suggesting that H3K4 methylation memory, which is normally associated with increased transcriptional activity, interfere with cell reprogramming and development of SCNT embryos (Liu W. et al., 2016; Hormanseder et al., 2017). In addition, modulation of H3K4 methylation levels with specific KDMs altered normal OET in mouse embryos (Dahl et al., 2016; Zhang et al., 2016). Among the different KDMs that regulate H3K4 methylation levels, loss of maternal KDM1A activity was shown to perturb OET (Wasson et al., 2016) and increase embryo development arrest (Ancelin et al., 2016). Moreover, overexpression of KDM1A in male germ cells disturbed progeny fetal development (Siklenka et al., 2015). KDM5B (also known as JARID1B) was also shown to regulate H3K4 methylation levels during OET (Dahl et al., 2016; Zhang et al., 2016), and its attenuation decreased porcine embryo development (Huang et al., 2015), and reduced SCNT efficiency in mice (Liu W. et al., 2016). However, the signaling pathways affected by KDM5B attenuation during embryo development were not characterized. In a previous study, we demonstrated that the relative mRNA expression of KDM5B and KDM5C (also known as JARID1C, and another KDM of H3K4), was dramatically increased during OET in porcine and bovine embryos (Glanzner et al., 2018), which suggested they are both involved in OET regulation in these species.

There is evidence from studies in somatic cells that changes in epigenetic marks modulate genome damage responses (GDR). Indeed, chromatin needs to be quickly remodeled at the sites of DNA double-strand breaks (DSBs) to allow specific repair factors access to act and repair the damaged DNA sites (Ayrapetov et al., 2014; Van and Santos, 2018; Nakamura et al., 2019; Sharma and Hendzel, 2019). Histone lysine demethylases of H3K4, including KDM1A, KDM5A, and KDM5B (Faucher and Wellinger, 2010; Li et al., 2014; Gong et al., 2017), as well as KDMs of H3K9, including KDM4B and KDM4D (Khoury-Haddad et al., 2014, 2015), were shown to be involved in the GDR process. There is also evidence that KDM5B regulates H3K4me levels near DSBs sites to facilitate the access of DNA repair enzymes, and its attenuation or pharmacological inhibition increased radioresistance in cancer cells (Bayo et al., 2018). The regulation of KDM5B in the context of GDR seems to be controlled by miRNAs (Mocavini et al., 2019). Not only KDMs but also histone methyltransferases (KMTs) of H3K4 (e.g., SET-2/SET1), appear to be required for protecting genome integrity during meiosis (Sommermeyer et al., 2013; Herbette et al., 2017; Adam et al., 2018). It has been proposed that the modulation of the H3K4me levels near DSBs sites is necessary for both gene repression and access of enzyme-complexes for DNA repair (Seiler et al., 2011; Li et al., 2014; Gong et al., 2017; Bayo et al., 2018).

The developmental competence of embryos, including mice (Wang et al., 2013), humans (Kort et al., 2016), and pigs (Bohrer et al., 2015, 2018; Dicks et al., 2017), is affected by DNA damage. Moreover, treatment with epigenetic modulators, such as histone deacetylates inhibitors, during embryo culture promoted DNA repair and improved embryo development (Bohrer et al., 2014). However, it has not been determined if the H3K4 methylation status mediate GDR during early embryo development. Therefore, this study was designed to investigate the role of KDM5B and KDM5C on the OET transition and GDR in early developing porcine embryos.

## Materials and Methods

Unless stated otherwise, all chemicals were purchased from Sigma Chemicals Company (Sigma-Aldrich; Oakville, ON, Canada).

### Oocyte Collection and *in vitro* Maturation (IVM)

Ovaries of prepubertal gilts were collected at a local slaughterhouse (Olymel S.E.C./L.P., Saint-Esprit, QC, Canada) and transported to the laboratory at 32°C in saline solution containing penicillin (100 UI/ml) and streptomycin (10 mg/ml). Cumulus-oocyte complexes (COCs) were aspirated from 3 to 6 mm follicles using a 10 mL syringe and 20-gauge needle and only COCs having a minimum of three cumulus cells layers and a homogeneous granulated cytoplasm were selected for IVM.

Groups of 30 COCs were matured at 38°C in 5% CO_2_ and 95% air for 22 h in 90 μl of maturation medium consisting of TCM-199 (Life technologies, Burlington, ON, Canada), supplemented with 20% of porcine follicular fluid, 1 mM dibutyryl cyclic adenosine monophosphate (dbcAMP), 0.1 mg/mL cysteine, 10 ng/mL epidermal growth factor (EGF; Life technologies), 0.91 mM sodium pyruvate, 3.05 mM D-glucose, 0.5 μg/mL LH (SIOUX Biochemical Inc., Sioux Center, IA, United States), 0.5 μg/mL FSH (SIOUX Biochemical Inc.), and 20 μg/mL gentamicin (Life technologies). COCs were transferred to the same IVM medium, but without LH, FSH, and dbcAMP, for an additional 20 to 22 h under the same conditions.

### *In vitro* Embryo Production

For *in vitro* fertilization (IVF), cumulus cells of matured oocytes were removed by vortexing in TCM-199 HEPES-buffered medium (Life Technologies) supplemented with 0.1% hyaluronidase. Denuded oocytes were washed three times in modified *Tris-*Buffered Medium (mTBM) (Abeydeera and Day, 1997), containing 2 mM caffeine and 0.2% bovine serum albumin (BSA, fatty acid free), and then fertilized in groups of 60–80 using 2 × 10^5^ sperm/ml in four-well plates with 500 μl media for 5 h.

For parthenogenetic activation (PA), matured oocytes with the first polar body were exposed to 15 μM ionomycin for 5 min in TCM-199 supplemented with 0.2% of BSA, and then cultured for 4 h in Ca^2+^ free porcine zygote medium (PZM-3) (Yoshioka et al., 2002) supplemented with 10 mM strontium chloride, 7.5 μg/mL cytochalasin B and 10 μg/mL cycloheximide.

For somatic cell nuclear transfer (SCNT), porcine fibroblast cells were cultured *in vitro* in Dulbecco’s Modified Eagle Medium/Nutrient Mixture F-12 Ham (DMEM-F12), supplemented with 10% FBS (Life Technologies) and antibiotics (10,000 U/mL penicillin and 10,000 μg/mL streptomycin) at 38°C in 5% CO_2_ and 95% air. Matured oocytes with a polar body were placed in TCM-199 supplemented with 0.2% BSA, 0.4 μg/mL demecolcine and 0.05 M sucrose for 60 min. This treatment resulted in a small protrusion in the ooplasmic membrane that contained the metaphase chromosomes. Oocytes were transferred to TCM-199 HEPES-buffered medium supplemented with 0.2% BSA, 20 μg/mL gentamicin, and 7.5 μg/mL cytochalasin B for 5–10 min, and then enucleated by removing the protruded chromatin and the first polar body. A nuclear donor cell was transferred into the perivitelline space of each enucleated oocyte, and then fused electrically using a single DC pulse of 32V for 70 μs. Electrofusion was performed in 0.28 M mannitol solution supplemented with 50 μM CaCl_2_, 100 μM MgSO_4_, and 0.1% BSA. Oocytes were then transferred to TCM-199 medium supplemented with 0.2% BSA for 1 h to allow cell fusion, and then activated as for PA.

After IVF, PA or SCNT, embryos were cultured in PZM-3 medium in a humidified atmosphere of 5% CO_2_ and 95% air at 38.5°C. At day 5 of culture, the medium was supplemented with 10% fetal bovine serum (FBS). Cleavage rates were evaluated at day 2 and blastocyst rates were assessed at day 7 of embryo culture.

### KDM5B and KDM5C Attenuation

Dicer-substrate interfering RNAs (DsiRNAs) were designed (Custom DsiRNA Design Tool) and synthetized by Integrated DNA Technologies (Windsor, ON, Canada). Specificity was confirmed by using the Basic Local Alignment Search Tool (BLAST; National Center for Biotechnology Information, Bethesda, MD, United States). Fertilized (IVF) or activated (SCNT and PA) oocytes were microinjected, using FemtoJet 4i (Eppendorf, Hamburg, Germany), with 10 pl of 25 μM diluted sense and antisense DsiRNAs targeting two unique sequences in the mRNA of KDM5B (si-KDM5B), KDM5C (si-KDM5C), both KDM5B and KDM5C (si-KDM5B + C), or control scrambled sequences (si-CT) (Supplementary Table S1). Microinjections were performed in TCM-199 HEPES-buffered medium supplemented with 2 mg/ml BSA (fatty acid free) and 20 mg/ml gentamicin. Knockdown efficiency was assessed by determining the relative mRNA abundance of *KDM5B* and *KDM5C* by real-time quantitative PCR (qPCR) at D3 and D5 after microinjection and KDM5B protein levels were assessed at D3 of embryo development.

### RNA Extraction and Reverse Transcription Quantitative PCR (RT-qPCR)

Total RNA was extracted from pools of 10–15 embryos at D3 and D5 of development using the PicoPure RNA Isolation Kit (Life Technologies). After extraction, RNA was treated with DNase I (Qiagen; Louiville, KY, United States), and then reverse transcribed using the SuperScript VILO cDNA Synthesis Kit (Life Technologies). RT qPCR reactions were performed in a CFX 384 real-time PCR system (BioRad, Hercules, CA, United States) using the advanced qPCR mastermix (Wisent Bioproducts, St-Bruno, QC, Canada). Primers were designed based on porcine (Supplementary Table S2) sequences available in GenBank, and synthesized by IDT (Windsor, ON, Canada). Samples were run in duplicates and the standard curve method was used to determine the relative abundance of mRNA for each gene. Relative mRNA expression was normalized to the mean abundance of the internal control gene *H2A*. All reactions had efficiency between 90 and 110%, *r*^2^ ≥ 0.98 and slope values from −3.6 to −3.1. Dissociation curve analyses were performed to validate the specificity of the amplified products.

### Immunofluorescence and Cell Counting Analyses

Developing embryos were fixed in 4% paraformaldehyde for 15 min and permeabilized with 1% Triton X-100 in PBS for 1 h at 37°C. Samples were incubated for 1 h at room temperature in blocking solution (3% BSA and 0.2% Tween-20 in PBS), then incubated overnight with primary antibodies anti- H3K4me1 (Abcam – ab8895, Toronto, ON, Canada), H3K4me2 (Millipore – 07-030, Billerica, MA, United States), H3K4me3 (Abcam – ab8580), H3K9me1 (Abcam – ab176880), H3K9me2 (Abcam – ab176882), H3K9me3 (Abcam – ab8898), KDM5B (Abcam – ab181089) or H2AX139ph (EMD Millipore – 05-636), according to manufacturer’s recommended dilutions in blocking solution. Negative control samples were incubated in blocking solution overnight in the absence of primary antibodies. Samples were then rinsed 3 times for 30 min each in blocking solution before incubation in the dark for 1 h at room temperature in the presence of the secondary antibodies, anti- mouse IgG Cy3-conjugated (Jackson ImmunoResearch Laboratories – 115-165-146, West Grove, PA, United States; 1:1000) or anti-rabbit IgG Alexa Fluor 488 (Abcam – ab150077; 1:2000) diluted in blocking solution. Embryos were then rinsed in blocking solution for 30 min, incubated for 20 min in 10 μg/ml 4,6-Diamidino-2-Phenylindole, Dilactate (DAPI), and rinsed once more in blocking solution for 30 min before mounting on slides in Mowiol. Slides were examined using a Nikon eclipse 80i microscope (Nikon), and images were captured at 200, 400 or 600 magnification using a Retiga 2000R monochromo digital camera (QImaging, Surrey, BC, Canada). The fluorescence intensity for H3K4me1, H3K4me2, H3K4me3, H3K9me1, H3K9me2, and KDM5B was quantified on each nucleus and corrected to the background in the cytoplasm of each embryo using the SimplePCI imaging software (Compix, Sewickley, PA, United States). The same software was used to determine the number of DSBs by counting the number of H2AX139ph foci ≥0.3 μm^3^ in each nucleus (Bohrer et al., 2018).

### Western Blot

Either si-KDM5B or si-CT were transfected into porcine fetal fibroblasts using the Neon^®^ transfection system (Life Technologies). Approximately 5 × 10^4^ cells were exposed to three electrical pulses of 1000V for 30 μs each in the presence of 10 nM si-KDM5B or si-CT. After 72 h, cells were lysed in Laemmli buffer (Bio-Rad, Mississauga, ON, Canada) containing protease inhibitor cocktail (G-Biosciences, St. Louis, MO, United States). Total lysate was size-fractionated by SDS-PAGE 10% gel and electro blotted onto nitrocellulose membranes (Bio-Rad). After blocking with 5% skim milk powder in *Tris-*buffered saline +0.1% Tween (pH 7.6), membranes were incubated overnight with primary anti- KDM5B (Abcam) or b-actin (Abcam) antibodies diluted 1:1000 and 1:5000 in blocking solution, respectively. Membranes were incubated for 1 h with horseradish peroxidase–conjugated secondary antibody anti-rabbit (Santa Cruz Biotechnology) diluted 1:10,000 in blocking solution, and then for 5 min with ImmunStar WesternC Chemiluminescent Kit (Bio-Rad). Images were captured using a ChemiDoc MP System (Bio-Rad), and bands were quantified using the Image Lab 3.0 software (Bio-Rad).

### Assessment of RNA Synthesis

Detection of RNA synthesis was performed using the Click-iT^®^ EU RNA Imaging Kits (Life Technologies). Embryos on D4 and D5 of development were incubated with 1 mM 5-ethynyluridine (EU; RNA synthesis) for 4 h, fixed in 4% paraformaldehyde, and then stained according to the manufacturer’s instructions and counterstained with DAPI. Samples were mounted on microscope slides and RNA synthesis was determined by counting the proportion of EU positive nuclei per embryo using a fluorescent microscope (Nikon eclipse 80i microscope).

### UV Light Exposure

To induce DNA damage, embryos at 36 h, D3, D5 or D7 of development post-PA were placed in 35 mm culture dishes (Corning, Tewksbury, MA, United States) containing 1 ml of PZM-3 medium placed in biologic safety cabinet (1300 Series Class II; Thermo Fisher Scientific, Waltham, MA, United States), and then exposed (UV+) or not (UV-) to UV light for 10 s. After UV treatment, embryos were washed and cultured for 30 min, 6 h or up to D7 and collected to extract mRNA for RT-qPCR analyses, assess DNA damage, or evaluate development to the blastocyst stage, depending on the experiment.

### Statistical Analysis

Data were analyzed using the JMP software (SAS Institute Inc., Cary, NC, United States). Differences in mRNA levels, cleavage, blastocyst, as well as RNA synthesis were analyzed by using the LSMeans Student *t-*test or Tukey-Kramer Honestly Significant Difference test for single or multiple variables, respectively. Data were tested for normal distribution using the Shapiro–Wilk test. Results are presented as means ± SEM, and *P* < 0.05 was considered statistically significant. All experiments were repeated at least 3 times.

## Results

### Attenuation of KDM5B and KDM5C Impaired Embryo Development

**FIGURE 1:**
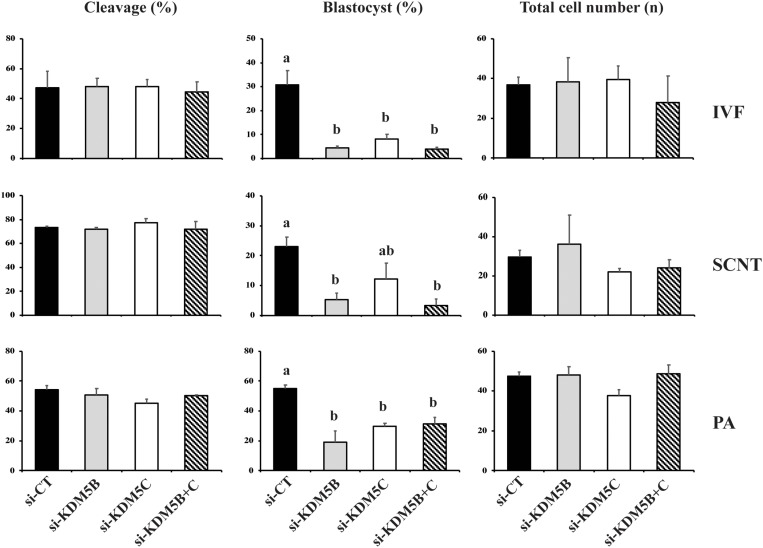
Effect of KDM5B and KDM5C attenuation on embryo development. Cleavage and blastocyst rates, and average number of cells in D7 blastocysts derived from control (si-CT) and attenuated (si-KDM5B, si-KDM5C or si-KDM5B + C) embryos produced by fertilization (IVF), nuclear transfer (SCNT), or parthenogenetic activation (PA). Si-CT: scramble sequence. Data are from three replicates for IVF (*n* = 120 oocytes/group/replicate) and PA (*n* = 100 oocytes/group/replicate), and from four replicates for SCNT (*n* = 40 oocytes/group/replicate). Different letters indicate statistical differences between treatments (*P* < 0.05).

In the first experiment, two DsiRNAs separately targeting KDM5B, KDM5C or both mRNAs were used to assess the effect of these KDMs on development of porcine embryos produced by IVF, SCNT or PA. Embryos produced by the three protocols were used to verify if the attenuation of these KDMs would differently affect embryos having only maternal chromatin (PA), having both maternal and paternal chromatins (IVF), or having both paternal and maternal chromatins, but derived from a differentiated cell (SCNT). Embryo cleavage was not altered by DsiRNA treatment (Figures 1, 10). However, development to the blastocyst stage was significantly reduced by attenuating KDM5B, or both KDM5B and KDM5C mRNAs in embryos produced by the three technologies. Attenuation of KDM5C mRNA significantly decreased blastocyst formation in IVF and PA embryos (Figures 1, 10). There was no additive effect on embryo development when both KDM5B and KDM5C mRNAs were attenuated compared to each KDM5B or KDM5C mRNA alone (Figure 1). The reduced proportion of embryos that developed to the blastocyst stage had similar number of cells compared to control embryos (Figure 1). The fact that embryos produced by the three different protocols were similarly affected by KDM5B and KDM5C attenuation indicate these KDMs are important for the establishment of the epigenetic program in porcine embryos, which is independent of the chromatin state inherited by the embryo, i.e., from gametes or somatic cells. Based on these results, embryos used in the subsequent studies were derived by PA.

The relative knockdown of *KDM5B* and *KDM5C* mRNAs was ∼75% on D3 and ∼60% on D5 embryos injected with either si-KDM5B or si-KDM5C compared to embryos injected with si-CT (Supplementary Figure S1). For KDM5B, the knockdown efficiency was confirmed at the protein level by immunofluorescence analysis in embryos and by western blotting in porcine fibroblast cells (Supplementary Figure S1). These results indicated that both KDM5B and KDM5C are required for normal development of porcine embryos. However, attenuation of both KDM5B and KDM5C mRNAs had no additive effect on decreasing embryo development, which suggests they regulate the same developmental processes. Therefore, the next objectives were to investigate the role of each of the two KDMs during early embryo development.

### Attenuation of KDM5B and KDM5C Altered mRNA Levels of Histone Demethylases

**FIGURE 2:**
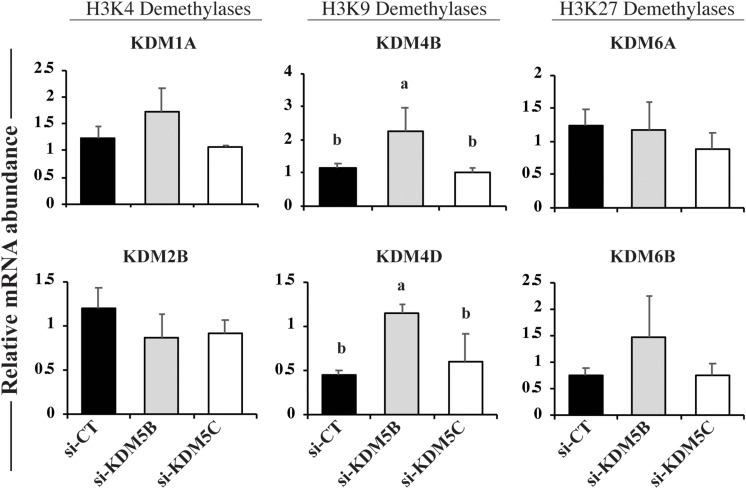
Relative mRNA levels of KDMs of H3K4, H3K9, and H3K27 on Day 3 PA embryos derived from oocytes injected with si-CT, si-KDM5B or si-KDM5C. Different letters indicate statistical differences between groups. Samples are from three replicates and RNA was extracted from pools of 10–15 embryos in each treatment and replicate. Different letters indicate statistical differences between treatments (*P* < 0.05).

In order to verify if mRNA attenuation of KDM5B and KDM5C, which are mainly involved in the regulation of H3K4 methylation levels, would result in the upregulation of other KDMs for compensatory effects, we quantified the relative mRNA abundance of KDMs of H3K4, H3K9, and H3K27. Embryos on Day 3 of development were used in this experiment because our previous studies revealed that first significant changes in the abundance of *KDMs* mRNA occurs at this early developmental stage in porcine embryos (Glanzner et al., 2018). Attenuation of *KDM5B* and *KDM5C* mRNAs did not affect the relative expression of *KDM1A* and *KDM2B* (H3K4 demethylases) mRNAs, as well as *KDM6A* and *KDM6B* (H3K27 demethylases) mRNAs (Figure 2). On the other hand, the relative mRNA abundance of *KDM4B* and *KDM4D* (H3K9 demethylases), was increased in KDM5B attenuated embryos, but similar to controls in KDM5C attenuated embryos (Figure 2). These observations are in line with findings from the next experiment, which revealed a decreased in H3K9 methylation levels in KDM5B attenuated embryos, but not in KDM5C attenuated embryos.

### Attenuation of KDM5B and KDM5C Modulated H3K4 and H3K9 Methylation

**FIGURE 3:**
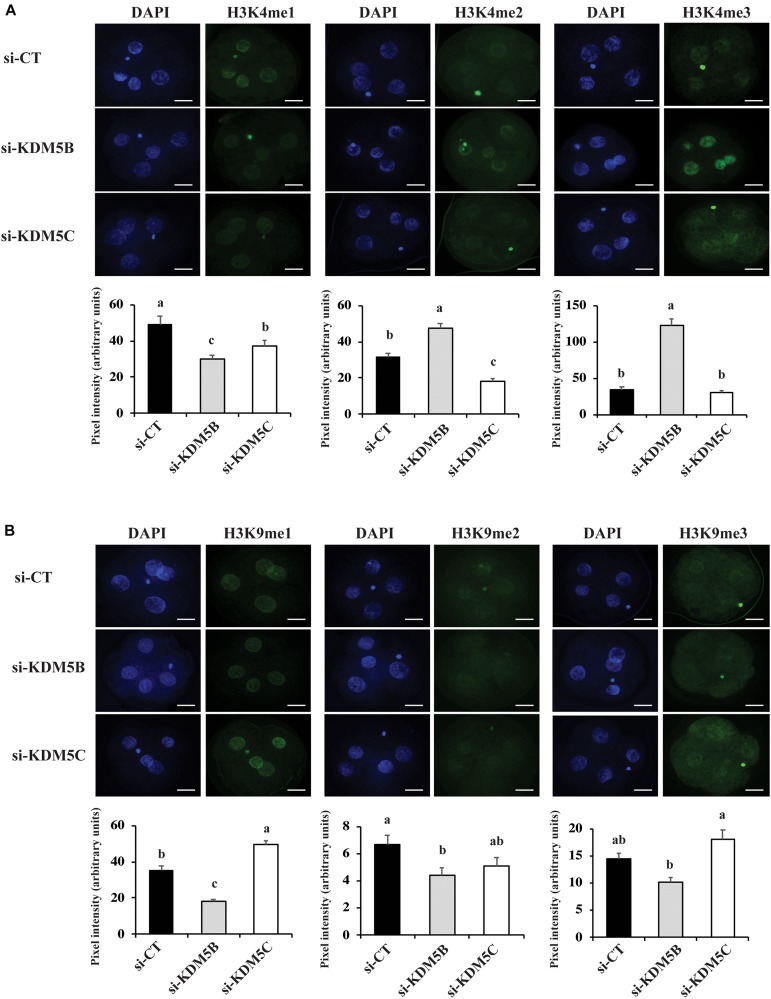
Detection of H3K4 and H3K9 methylation by immunofluorescence on Day 3 PA embryos derived from oocytes injected with si-CT, si-KDM5B or si-KDM5C. **(A)** Representative pictures and quantification of the pixel intensity for H3K4-me1, me2 and me3. **(B)** Representative pictures and quantification of the pixel intensity for H3K9-me1, me2, and me3. Three independent replicates were performed and 10–15 embryos were used to quantify pixel intensities for each epigenetic mark. Different letters indicate statistical significance between treatments for each epigenetic mark (*P* < 0.05). Scale bars represent 25 μm.

**FIGURE 4:**
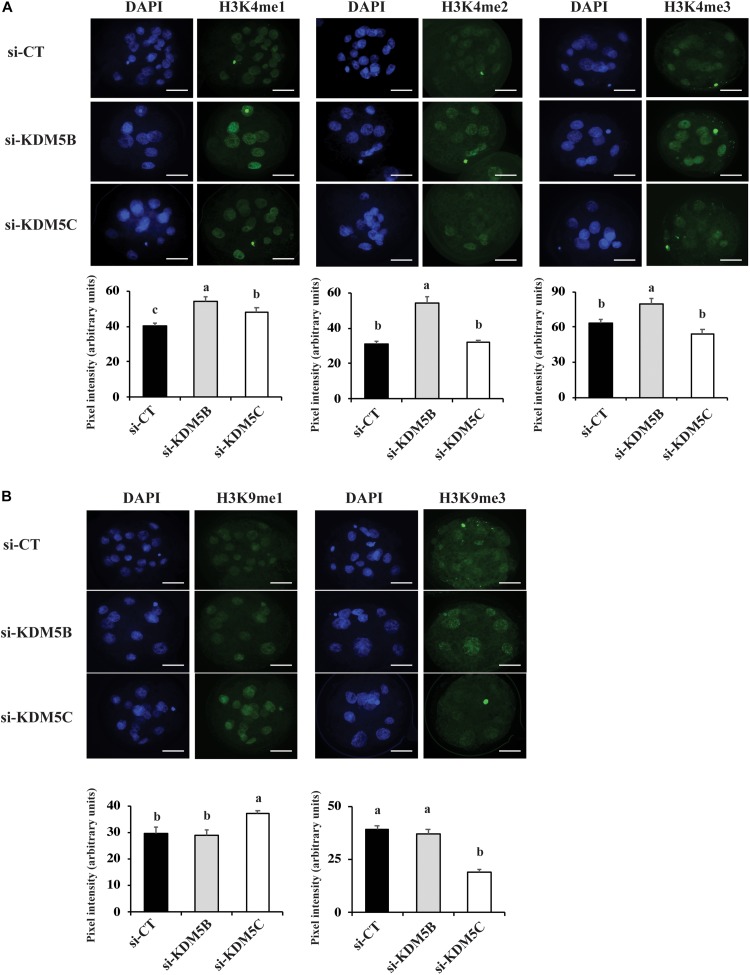
Detection of H3K4 and H3K9 methylation by immunofluorescence on Day 5 PA embryos derived from oocytes injected with si-CT, si-KDM5B or si-KDM5C. **(A)** Representative pictures and quantification of the pixel intensity for H3K4-me1, me2 and me3 staining. **(B)** Representative pictures and quantification of the pixel intensity for H3K9me1, me2 and me3 staining. Three independent replicates were performed and 4–6 embryos were used to quantify pixel intensities for each epigenetic mark. Different letters indicate statistical significance between treatments for each epigenetic mark (*P* < 0.05). Scale bars represent 25 μm.

To investigate if attenuation of *KDM5B and KDM5C* mRNAs alters histone methylation levels, we assessed mono(me1)-, di(me2)-, and tri(me3)-methylation levels of H3K4 and H3K9 on D3 and D5 embryos. On D3 embryos, attenuation of *KDM5B* increased H3K4me2 and H3K4me3 levels (Figures 3A, 10), but decreased H3K4me1, H3K9me1 and H3K9me2 levels (Figures 3A,B), whereas *KDM5*C attenuation decreased H3K4me1 and H3K4me2 (Figure 3A), but increased H3K9me1 levels (Figures 3B, 10) compared to control embryos. On D5 embryos, *KDM5B* attenuation increased H3K4me1, H3K4me2, and H3K4me3 levels (Figures 4A, 10), whereas *KDM5C* attenuation increased H3K4me1 and H3K9me1 (Figures 4A,B, 10), and decreased H3K9me3 levels (Figure 4B) compared to control embryos. These findings suggest that KDM5B mainly regulates H3K4 methylation, while KDM5C mainly regulates H3K9 methylation during early development of porcine embryos. The immunofluorescent signal for H3K9me2 was either absent or barely detectable on D5 embryos and, therefore, it was not quantified.

### KDM5B and KDM5C Attenuation Perturbed Embryo Genome Activation

**FIGURE 5:**
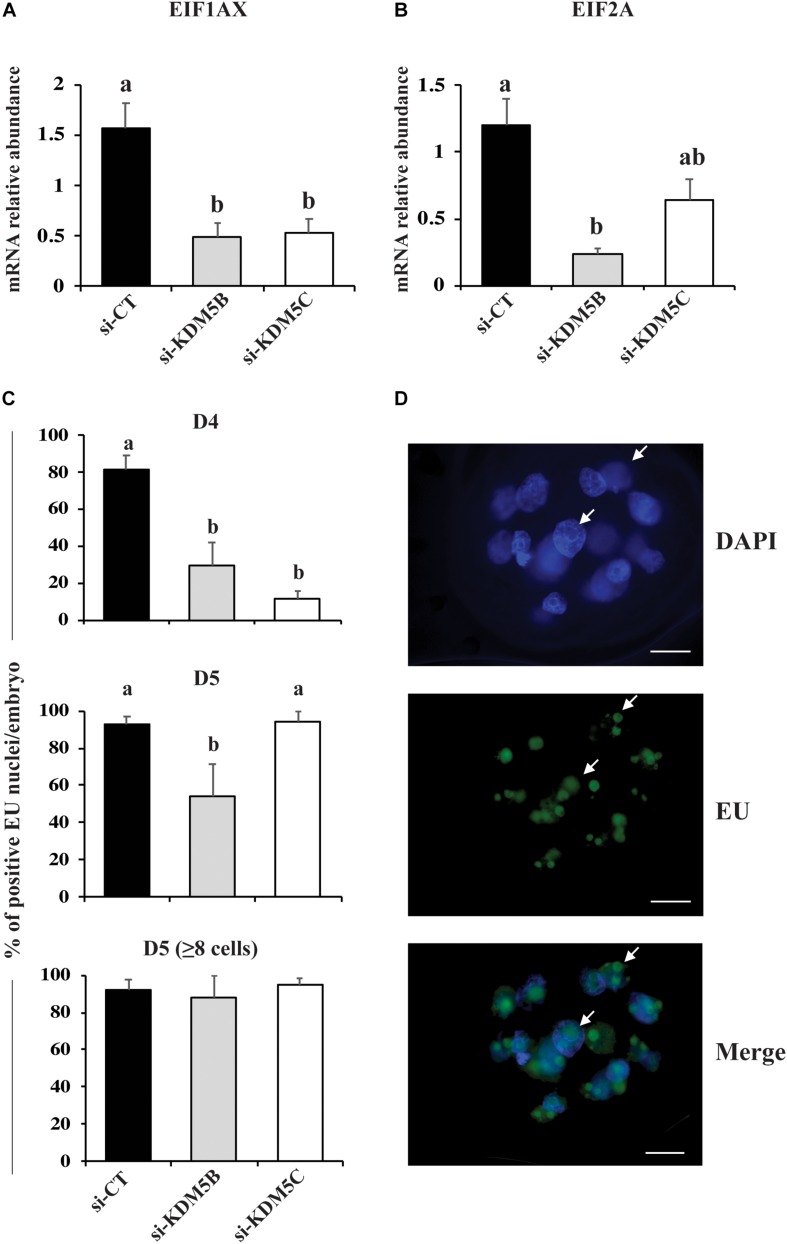
Detection of genome activation markers in PA embryos derived from oocytes injected with si-CT, si-KDM5B or si-KDM5C. Relative mRNA levels of **(A)** *EIF1AX* and **(B)** *EIF2A* on Day 3 embryos. Samples are from three replicates and RNA was extracted from pools of 10–15 embryos in each treatment and replicate. **(C)** Proportion of nuclei with positive fluorescent signal for EU incorporation on Day 4 and Day 5 (total or those with >8-cells) embryos. Samples are from three replicates and 5–8 embryos were used for quantification of EU incorporation in each treatment and developmental stage. **(D)** Representative images showing EU incorporation on a Day 5 embryo (arrows indicate fluorescent signal for EU incorporation). Different letters indicate statistical differences between treatments (*P* < 0.05). Scale bars represent 25 μm.

**FIGURE 6:**
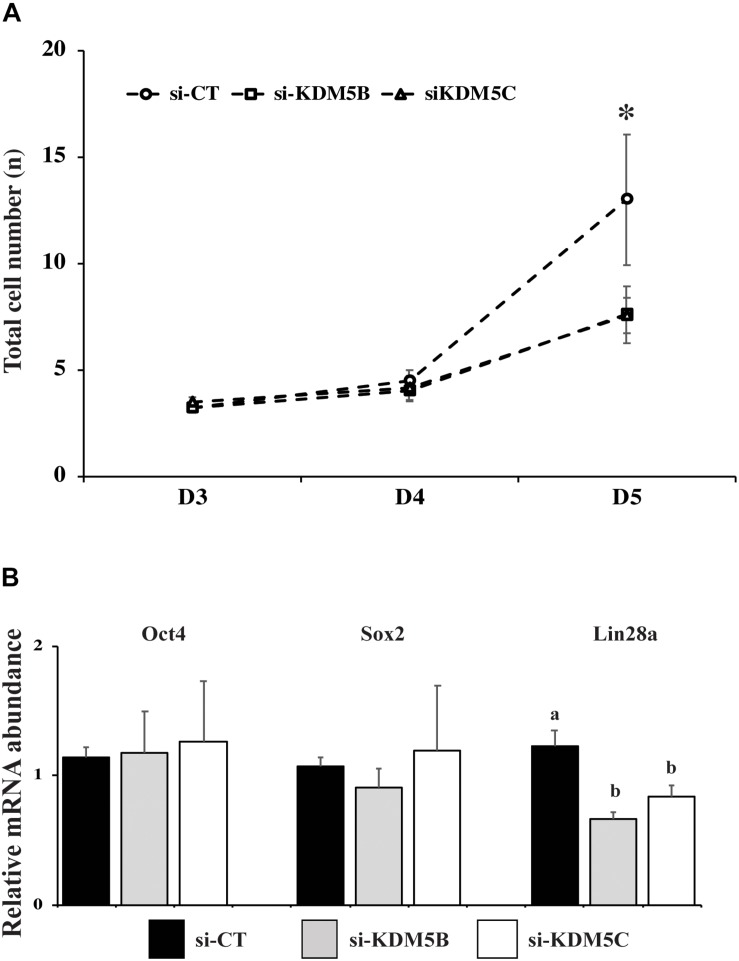
Number of cells and expression of pluripotency-related genes in PA embryos derived from oocytes injected with si-CT, si-KDM5B or si-KDM5C. **(A)** Average number of cells on Day 3, Day 4, and Day 5 embryos. (^∗^) indicate statistical differences between si-CT and si-KDM5B or si-KDM5C groups. Embryos were collected from three replicates and 10–15 embryos were analyzed for each developmental stage in each treatment. **(B)** Relative mRNA expression of OCT4, SOX2 and Lin28a in Day 5 embryos. Different letters indicate statistical differences between treatments. Samples are from three replicates and RNA was extracted from pools of 10–15 embryos in each treatment and replicate. (^∗^) indicates indicate statistical difference between si-CT versus si-KDM5B and si-KDM5C (*P* < 0.05).

Since *KDM5B* and *KDM5C* attenuation severely hampered embryo development, we investigated if this was in consequence of abnormal EGA. Quantification of the relative mRNA levels of *EIF1AX* and *EIF2A*, important indicators of EGA in porcine embryos, revealed that either *KDM5B* or *KDM5C* attenuation prevented mRNA expression of these genes on D3 embryos (Figures 5A,B). Similarly, the immunofluorescent signal for EU incorporation, which indicates new RNA synthesis (Figure 5D), revealed that attenuation of *KDM5B* and *KDM5C* mRNA significantly decreased the proportion of nuclei with EU incorporation on D4 embryos (Figure 5C). On D5 embryos, the proportion of nuclei with EU incorporation remained lower in *KDM5B* attenuated embryos, but not in *KDM5C* attenuated embryos (Figure 5C). However, when only embryos having ≥8 cells on D5 were analyzed, there was no difference in the proportion of nuclei with positive signal for new RNA synthesis (Figure 5C), which suggests a delayed development in *KDM5B* attenuated embryos. Indeed, the total cell number was decreased in either *KDM5B* or *KDM5C* attenuated-embryos at D5 of development (Figures 6A, 10). In addition, the relative mRNA abundance of *Lin28*, which regulates cell differentiation and pluripotency, was lower in either *KDM5B* or *KDM5C* attenuated-embryos compared to control D5 embryos (Figure 6B). These findings indicate that KDM5B and KDM5C are required for normal EGA transition and development beyond the 4-cell stage in porcine embryos.

### KDM5B and KDM5C Attenuation Affected DNA Repair

**FIGURE 7:**
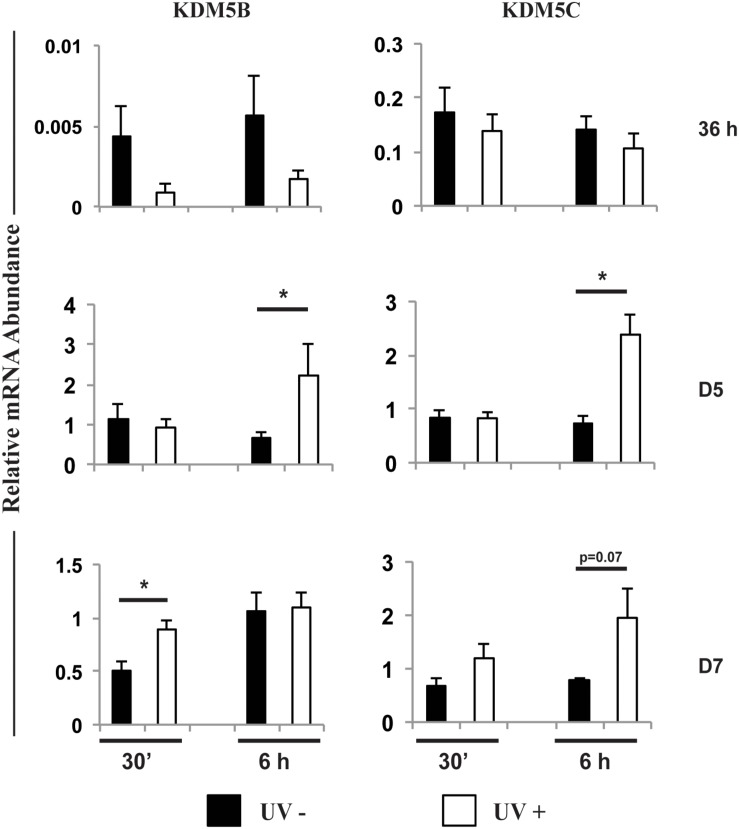
Relative mRNA abundance of *KDM5B* and *KDM5C* in embryos exposed (UV+) or not (UV-) to UV radiation for 10 s at 36 h, Day 5 or Day 7 post-PA. Samples were collected at 30 min or 6 h after UV exposure. (*) indicate statistical differences between UV+ and UV- embryos. Samples are from three replicates and RNA was extracted from pools of 10–15 embryos in each treatment and time point.

To further investigate the roles of KDM5B and KDM5C on embryo development, we analyzed the effect of these KDMs on DNA damage response. First, embryos were UV-irradiated for 10 s either at 36 h, D5 or D7 post-activation, and samples were collected at 30 min or 6 h after UV treatment. The relative mRNA levels of *KDM5B* and *KDM5C* were similar between control and UV-irradiated embryos at 36 h after activation (Figure 7). However, both *KDM5B* and *KDM5C* mRNA levels were increased on D5 embryos at 6 h after UV treatment compared to control embryos (Figure 7). *KDM5B* mRNA levels were also increased on D7 embryos at 30 min after UV exposure compared to control embryos (Figure 7). To confirm the effect of UV treatment on the activation of DNA repair pathways, the relative mRNA abundance of *ATM*, *RAD5*1 and *BRCA1* were assessed. Exposure to UV reduced *ATM* and *BRCA1* mRNA levels in embryos treated at 36 h after activation (Supplementary Figure S2), likely reflecting an accelerated translation to promote DNA repair in this transcriptionally incompetent pre-EGA stage embryos. However, in transcriptional competent post-EGA stage embryos, UV irradiation increased the mRNA abundance of *ATM*, *RAD51* and *BRCA1* on D5 embryos, and *BRCA1* on D7 embryos at 6 h after UV exposure (Supplementary Figure S2), confirming the upregulation of DNA repair genes in response to the UV treatment. These results suggested that KDM5B and KDM5C are involved in the regulation of DNA damage repair during early embryo development.

**FIGURE 8:**
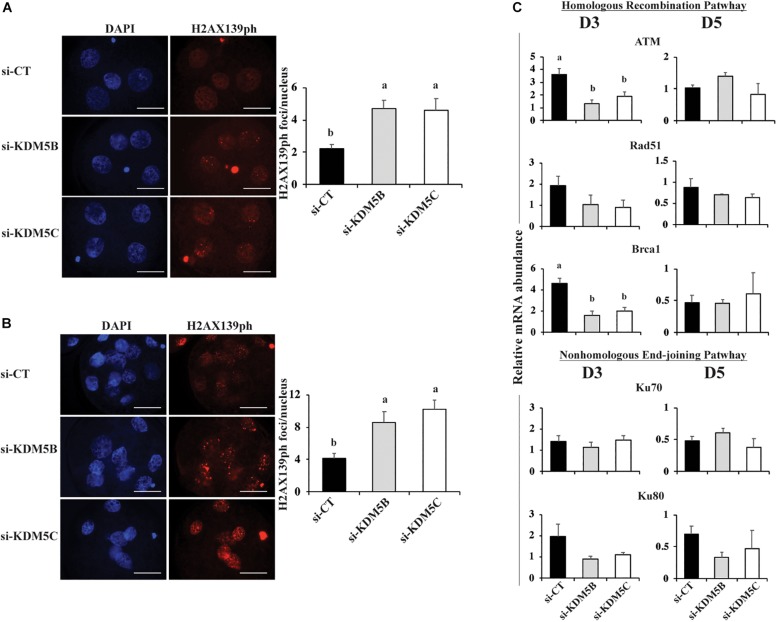
DNA damage and mRNA expression of DNA repair genes in PA embryos derived from oocytes injected with si-CT, si-KDM5B or si-KDM5C. **(A)** Representative pictures and average number of H2AX139ph foci in each nucleus of Day 3 **(A)** and Day 5 **(B)** embryos. Three independent replicates were performed and 15–20 embryos were analyzed per treatment and developmental stage for quantification H2AX139ph foci. Scale bars represent 25 μm. **(C)** Relative mRNA abundance of genes involved in the homologous recombination (ATM, RAD51, and BRCA1) and the non-homologous end-joining (Ku70 ad Ku80) pathways of DNA repair on Day 3 and Day 5 embryos. Samples are from three replicates and RNA was extracted from pools of 10–15 embryos in each treatment and developmental stage. Different letters indicate statistical differences between treatments (*P* < 0.05).

Next, we investigated if attenuation of *KDM5B* and *KDM5C* affect DNA damage response. We found that attenuation of either *KDM5B* or *KDM5C* mRNA increased ∼2-fold the number of DNA double strand breaks (DSBs) per nuclei, as assessed by the number of H2AX139ph foci, in both D3 (Figure 8A) and D5 (Figures 8B, 10) embryos compared to controls. To investigate if this was caused by altered regulation of DNA repair genes, the relative mRNA expression of genes involved in the homologous recombination (HR) and non-homologous end joining (NHEJ) pathways of DNA repair were evaluated on D3 and D5 embryos. Attenuation of *KDM5B* and *KDM5C* decreased mRNA levels of *ATM* and *BRCA1*, which are involved in the HR repair pathway, on D3 embryos (Figure 8C). However, attenuation of *KDM5B* and *KDM5C* did not significantly affect the mRNA abundance of genes involved in the NEHJ repair pathway (Figure 8C). These findings indicated that KDM5B and KDM5C regulate DNA repair in early developing porcine embryos, likely through modulation of genes involved in the HR repair pathway.

**FIGURE 9:**
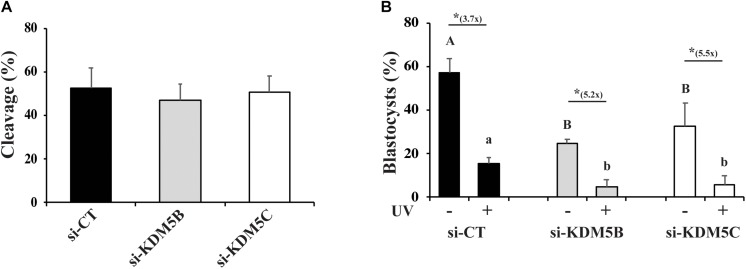
Effect of KDM5B and KDM5C attenuation on development of embryos exposed to UV radiation for 10 s. **(A)** Cleavage rates of oocytes injected with si-CT, si-KDM5B or si-KDM5C. **(B)** Development to the blastocyst stage of embryos derived from oocytes injected with si-CT, si-KDM5B or si-KDM5C and exposed (UV+) or not (UV-) to UV radiation on Day 3 of development. Uppercase letters indicate statistical differences between groups in UV- embryos, and lowercase letters indicate statistical differences between groups in UV+ embryos (*P* < 0.05). (*) Indicates indicate statistical difference between UV- and UV + embryos within the same group (*P* < 0.05). Data are from three individual replicates with at least 75 embryos in each treatment. 25–30 embryos each.

Lastly, we tested the effect of UV irradiation on development of *KDM5B* and *KDM5C* attenuated embryos. For that, control, *KDM5B-* and *KDM5C-* attenuated embryos were UV-treated for 10 s on D3 of development and then cultured until D7 to assess blastocyst rates. As expected, *KDM5B* and *KDM5C* attenuation did not affect embryo cleavage (Figure 9A), and UV-irradiation significantly decreased embryo development to the blastocyst stage (Figure 9B). Remarkably, development to the blastocyst was almost completely suppressed by either *KDM5B* or *KDM5C* attenuation in UV-irradiated embryos (Figure 9B). This further supports a relevant role of KDM5B and KDM5C in the regulation DNA damage repair in early developing porcine embryos.

**FIGURE 10:**
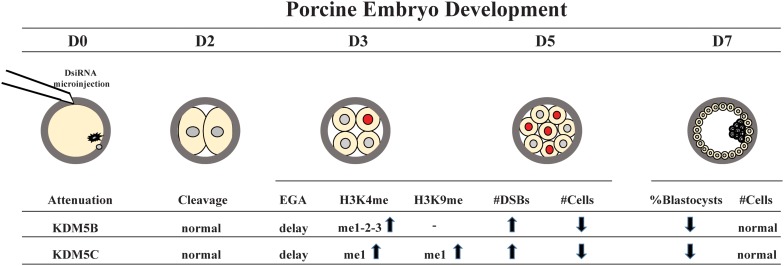
Schematic model of KDM5B and KDM5C roles during early embryo development. KM5B and KDM5C attenuation do not affect embryo cleavage but cause a delay in embryo development and impair EGA transition probably through modulation of H3K4 and H3K9 methylation levels. In addition, KDM5B and KDM5C interfere with DNA damage repair, mainly affecting the HR repair pathway, thus leading to an increase in DNA DSBs and genome instability.

## Discussion

After fertilization, a number of epigenetic changes are required to setup and modulate the embryo developmental program. Activation of transcriptional activity, cell lineage commitment and DNA damage repair are among the critical events regulated by epigenetic modifications in early developing embryos (Ostrup et al., 2013; Bohrer et al., 2015; Dahl et al., 2016; Dicks et al., 2017; Ding et al., 2017; Eckersley-Maslin et al., 2018; Rissi et al., 2019). Both maternal and paternal derived chromatins must be epigenetically programed after fertilization to make embryos capable to pursue their normal developmental course (Siklenka et al., 2015; Dahl et al., 2016; Teperek et al., 2016). A variety of epigenetic regulators such as erasers (e.g., KDM1A, KDM4D, KDM7A) and writers (e.g., SETDB1, SUV39H1/H2, G9a) are necessary to properly implement the epigenetic program in embryos (Jullien et al., 2014; Matoba et al., 2014; Golding et al., 2015; Siklenka et al., 2015; Dahl et al., 2016; Eymery et al., 2016; Liu W. et al., 2016; Liu X. et al., 2016; Zhang et al., 2016; Dumdie et al., 2018; Zylicz et al., 2018; Rissi et al., 2019). However, much remains to be done to uncover the function and interaction of each epigenetic regulator. This includes a number of different KDMs, the enzymes responsible for demethylating lysine residues in histones, whose expression pattern was shown to dramatically change in early developing embryos of different species (Dahl et al., 2016; Liu W. et al., 2016; Zhang et al., 2016; Hormanseder et al., 2017; Glanzner et al., 2018). In this study, we chose to investigate the role of KDM5B and KDM5C, both known to act on H3K4me, during porcine embryo development because our previous studies revealed that their expression increased markedly, but transiently around the OET stage in porcine embryos (Glanzner et al., 2018), and because KDM5B attenuation was shown to reduce development of porcine embryos (Huang et al., 2015).

First, we found that attenuation of KDM5B or KDM5C similarly decreased embryo development rates, but attenuation of both had no synergistic effect. This was observed in embryos produced by IVF, SCNT and PA, which not only suggested that the two KDMs regulate similar functions but also indicated a critical role of both KDM5B and KDM5C in the programming of porcine early embryo development. To investigate their roles, we started by assessing if H3K4me and H3K9me methylation levels were similarly affected by KDM5B and KDM5C attenuation on D3 and D5 embryos. Although both KDM5B and KDM5C, are recognized to act on H3K4 (Vallianatos and Iwase, 2015; Hyun et al., 2017), we observed significant increase in the overall H3K4me levels (H3K4me1 on D5, H3K4me2 on D3 and D5, and H3K4me3 on D3 and D5) only in KDM5B attenuated embryos. In KDM5C attenuated embryos, only H3K4me1 levels on D5 embryos were increased. On the other hand, KDM5C attenuation increased H3K9me1 on D3 and D5, while KDM5B attenuation did not increase H3K9me marks. These findings suggest that KDM5C may regulate both H3K4me and H3K9me levels in early developing porcine embryos, while KDM5B seems to regulate H3K4me only. There is evidence from previous studies in neuronal cells suggesting that KDM5C is involved in regulating H3K4me levels directly, but it can also indirectly regulate H3K9me levels by interacting with histone methylases (Iwase et al., 2007; Tahiliani et al., 2007). We also observed a decrease in H3K9me levels (H3K9me1-2) in KDM5B attenuated embryos on D3 of development, which was correlated with an increase in the relative mRNA expression of the H3K9 demethylases, *KDM4B* and *KDM4D*. On the other hand, there was a decrease in H3K4me levels (H3K4me1-2-3) in KDM5C attenuated embryos on D3 of development, which was not correlated with the mRNA expression of two H3K4 demethylases (*KDM1A* and *KDM2B*) evaluated in this study. It is possible that KDM5C attenuation either increased the expression of other KDMs of H3K4 or decreased the expression of KMTs of H3K4. There is recent evidence suggesting a cross-talk between H3K4 and H3K9 lysine modifying enzymes (Li et al., 2008; Mishra et al., 2018) and a role of H3K4/H3K9 bivalent domains in the regulation of cell differentiation (Matsumura et al., 2015).

In order to determine which vital embryo function leading to poor embryo development was perturbed by KDM5B and KDM5C attenuation, the next goal was to investigate possible effects on OET transition by assessing EU incorporation and mRNA abundance of *EIF1AX* and *EIF2A*, which are important markers of EGA in porcine embryos. *EIF1AX* expression is correlated with the embryo potential for development (Magnani et al., 2008). A marked decrease in both EU incorporation and the relative mRNA abundance of *EIF1AX* and *EIF2A* was detected in KDM5B and KDM5C attenuated embryos compared to controls. These results indicate that both KDM5B and KDM5C are involved in the regulation of EGA in porcine embryos and are in line with studies in mice suggesting that a broad lysine demethylation, including H3K4, is required in the embryo for the proper initiation of its transcriptional activity (Dahl et al., 2016; Zhang et al., 2016). We have also found a delay in the embryo development, as evidenced by the lower number of cells on D5 embryos compared to controls, which further suggests a delay in the EGA transition and expression of genes important for embryo development in KDM5B and KDM5C attenuated embryos. Analysis of mRNA abundance of three candidate genes involved in cell pluripotency and lineage specification (*OCT4*, *SOX2* and *Lin28a*) on D5 embryos revealed a significant decrease of *Lin28a*, but not *OCT4* and *SOX2*, in KDM5B and KDM5C attenuated embryos. Although Lin28a’s role on porcine embryo development remains not fully characterized, it is known to regulate pluripotency (Fukuda et al., 2017, 2019), as well as development, metabolism and germ cell specification in other species (Faas et al., 2013; Shyh-Chang and Daley, 2013; Ouchi et al., 2014; Tsialikas and Romer-Seibert, 2015). This suggests that KDM5B and KDM5C attenuation may have long term consequences on cell differentiation and development. Nonetheless, the total cell number and proportion of cells in the ICM and trophoblast in KDM5B and KDM5C attenuated embryos that developed to the blastocyst stage were similar to controls.

The last goal in this study was to evaluate if KDM5B and KDM5C attenuation affect embryo capacity to regulate DNA damage repair. There is evidence from studies in somatic cells that histone demethylases participate in DNA damage repair (Faucher and Wellinger, 2010; Khoury-Haddad et al., 2014, 2015; Li et al., 2014; Gong et al., 2017), a function that is likely mediated through chromatin remodeling near the damaged sites to facilitate access of DNA repair factors (Seiler et al., 2011; Li et al., 2014; Gong et al., 2017; Bayo et al., 2018). In porcine embryos, attenuation of KDM5B increased DNA fragmentation (Huang et al., 2015), and attenuation of WRD5, a component of the MLL complexes involved in H3K4 methylation, increased DNA damage (Ding et al., 2017). This suggests that KDMs’ actions are also important for DNA repair in embryos. Findings in this study revealed that mRNA abundance of both *KDM5B* and *KDM5C* was increased in transcriptional competent (day 5) embryos exposed to UV radiation to induce DSBs compared to control embryos. On the other hand, attenuation of KDM5B and KDM5C mRNA significantly increased DSBs, as assessed by the number of H2AX139ph foci. In addition, attenuation of either KDM5B or KDM5C in UV-treated embryos almost completely suppressed development to the blastocyst stage. These results provide solid evidence that both KDM5B and KDM5C are involved in the DNA damage response in porcine embryos. The fact that KDM5B and KDM5C attenuation also decreased development to blastocysts stage in non-UV exposed embryos may also be in consequence of accumulation of DSBs. This is supported by our previous findings revealing that late cleaving and less developmentally competent embryos have increased incidence of DSBs (Bohrer et al., 2015; Dicks et al., 2017).

## Conclusion

In conclusion, findings from this study revealed that both KDM5B and KDM5C are important for normal development of porcine embryos as they are involved in the embryo transcription activation and DNA damage response.

## Data Availability Statement

The datasets generated for this study are available on request to the corresponding author.

## Author Contributions

WG, LA, and VB conceptualized the experiments. WG was responsible for conducting the experiments, data analysis, and writing the manuscript. KG, VR, MM, RL, LC, ND, and HB assisted with embryo production experiments, data collection and analysis. LA and VB participated in data analysis and edited the manuscript. All authors contributed to the manuscript final review and editing.

## Conflict of Interest

The authors declare that the research was conducted in the absence of any commercial or financial relationships that could be construed as a potential conflict of interest.
