# Supplementary material for: Histone Lysine Demethylases KDM5B and KDM5C Modulate Genome Activation and Stability in Porcine Embryos
Source: Front Cell Dev Biol. 2020 Mar 10;8:151. doi: 10.3389/fcell.2020.00151 (PMC7076052; doi:10.3389/fcell.2020.00151)
Supplement: Supplementary file 1 [file Data_Sheet_1.PDF]

Table S1. DsiRNAs used for knockdown experiments.

Target	Sense	Antisense
KDM5B#1	CUGAUUACUUCAACAUGCCAGUCCA	UGGACUGGCAUGUUGAAGUAAUCAGAU
KDM5B#2	AUGUACAGCAUUAAAAUCUUACCTC	GAGGUAAGAUUUUAAUGCUGUACAUGA
KDM5C#1	CAAGAAGAAUUCUUGCUACACUCTG	CAGAGUGUAGCAAGAAUUCUUCUUGAG
KDM5C#2	UACAAGUGGUUGGAACCUGAAUGTG	CACAUUCAGGUUCCAACCACUUGUAGC
Negative control	CGUUAAUCGCGUAUAAUACGCGUA	AUACGCGUAUUAUACGCGAUUAACGA

Table S2. List of primers and accession number of analyzed genes

Gene	Forward Primer	Reverse primer	Accession Number or reference
H2A	GGTGCTGGAGTATCTGACCG	GTTGAGCTCTTCGTCGTTGC	Glanzner et al., 2018
Kdm5b	GACGTGTGCCAGTTTTGGAC	TCGAGGACACAGCACCTCTA	Glanzner et al., 2018
Kdm5c	GGCATGGTCTTCTCAGCCTT	TGAGGGTACCCCATACCAGG	Glanzner et al., 2018
Kdm2b	CTTCAGCTACCTCAGCCACC	TTATCACAGCACCAGCGGTT	Glanzner et al., 2018
Kdm1a	TCGTGTGGGTGGAAGAGTTG	CTTGTTTGCTGACCACAGCC	Glanzner et al., 2018
Kdm4b	GCGACTAGCAATCGGCTTCT	GGTCATCTTGTGCCGAAGGA	Glanzner et al., 2018
Kdm4d	CATACTGGAAAACGCGCCTG	CCACATGCCGAAGTACAGGT	XM_003129775.5
Kdm6a	AGCTTTTGTCGAGCCAAGGA	GCATTGGACAAAGTGCAGGG	Glanzner et al., 2018
Kdm6b	GGGAGACTATCAGCGCCTTC	AGCGGTACACAGGGATGTTG	Glanzner et al., 2018
Oct4	GCCAAGCTCCTAAAGCAGAAG	GCCAAGCTCCTAAAGCAGAAG	NM_001113060.1
Sox2	AACCAGAAGAACAGCCCAGAC	CTCCGACAAAAGTTTCCACTCG	NM_001123197.1
Lin28a	TTCTGCATTGGGAGCGAGAG	GCAGTTTGCATTCCTTGGCA	NM_001123133.1
Eif1ax	ACACCTCCCCGATAGGAGTC	TTGAGCACACTCTTGCCCAT	NM_001243218.1
Eif2a	AGGAGCGTCCTACTATGGGG	TGGCTGGCATGAAGCCATAA	NM_001319043.1
ATM	GCACCAATCCAGTGTAGGCT	AATAGCAGCCAAGGACACCC	NM_001123080.1[a]
Rad51	CGGTGGAAGAGGAGAGCTTTG	TTTAGCTGCCTCGGTCAGAAT	NM_001123181.1[b]
Brca1	CTTCTGTGGTGAAGGACCCC	TCACATGGAAGCCACTGTCC	XM_005656956.3[c]
Ku70	TTCAAGCCCTTGGGAATGCT	CTTGGTGAGCAGAGCAGTGA	NM_001190185.1[d]
Ku80	TTCCTGAGAGCCCTTCGAGA	TTTGGGCTTCCTCGACTGTG	XM_003133649.5[e]

[a]Homologous region between 15 transcripts: NM_001123080.1; XM_021101922.1; XM_021101923.1; XM_021101924.1; XM_021101925.1; XM_021101926.1; XM_021101927.1; XM_021101928.1; XM_021101929.1; XM_021101931.1; XM_021101932.1; XM_021101933.1; XM_021101934.1; XM_021101935.1; XM_021101936.1.
[b]Homologous region between two transcripts: NM_001123181.1; XM_021082812.1.
[c]Homologous region between 18 transcripts: XM_021066931.1; XM_021066926.1; XM_021066924.1; XM_021066932.1; XM_021066933.1; XM_021066936.1; XM_021066927.1; XM_021066928.1; XM_021066929.1; XM_021066930.1; XM_021066925.1; XM_021066935.1; XM_021066934.1; XM_005656957.3; XM_005656958.3; XM_005656956.3; XM_013989935.2; XM_003358030.4.
[d]Homologous region between four transcripts: NM_001190185.1; XM_021090940.1; XM_021090941.1; XM_021090942.1.
[e]Homologous region between three transcripts: XM_003133649.5; XM_013984470.2; XM_005672177.3

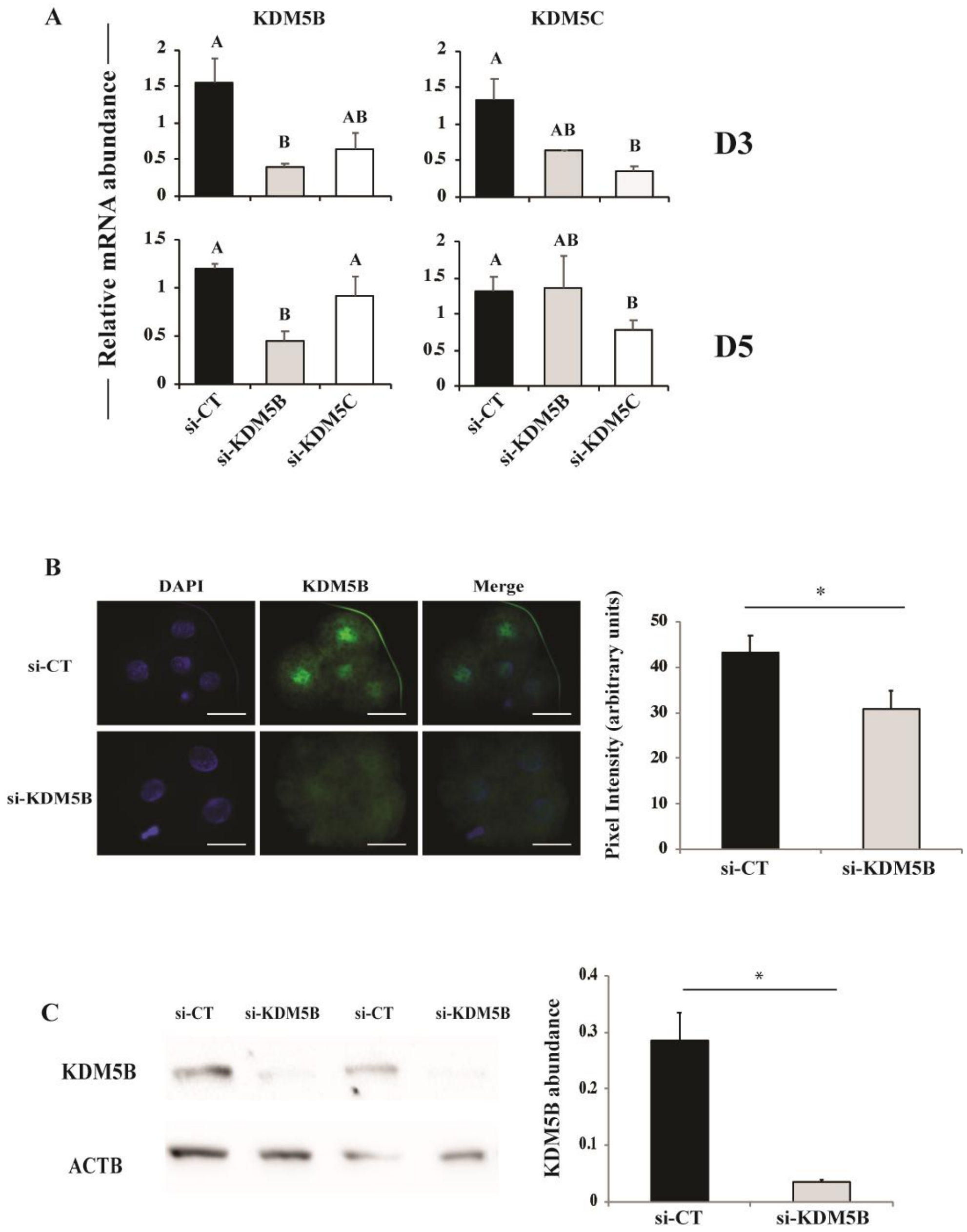

Fig. S1. KDM5B and KDM5C attenuation after DsiRNA treatment of oocytes and somatic cells. (A) Relative mRNA levels of *KDM5B* and *KDM5C* at Day 3 and Day 5 of development in PA embryos derived from oocytes injected with si-CT, si-KDM5B or si-KDM5C. Different letters indicate statistical differences between treatments for each gene and stage of embryo development ($P<0.05$). Samples are from three replicates and RNA was extracted from pools of 10-15 embryos in each treatment and replicate. (B) Representative pictures and quantification of the immunofluorescent signal for KDM5B on Day 3 embryos derived from oocytes injected with si-CT, si-KDM5B. (*) Indicates indicate statistical difference between groups ($P<0.05$). Samples are from three replicates and 20 embryos from each treatment were used for quantification of the immunofluorescent intensity. (C) Representative immunoblotting picture and quantification of KDM5B protein in porcine fibroblast cells at 72 h after electroporation with si-CT, si-KDM5B. (*) Indicates indicate statistical difference between groups ($P<0.05$). Four independent replicates were performed.

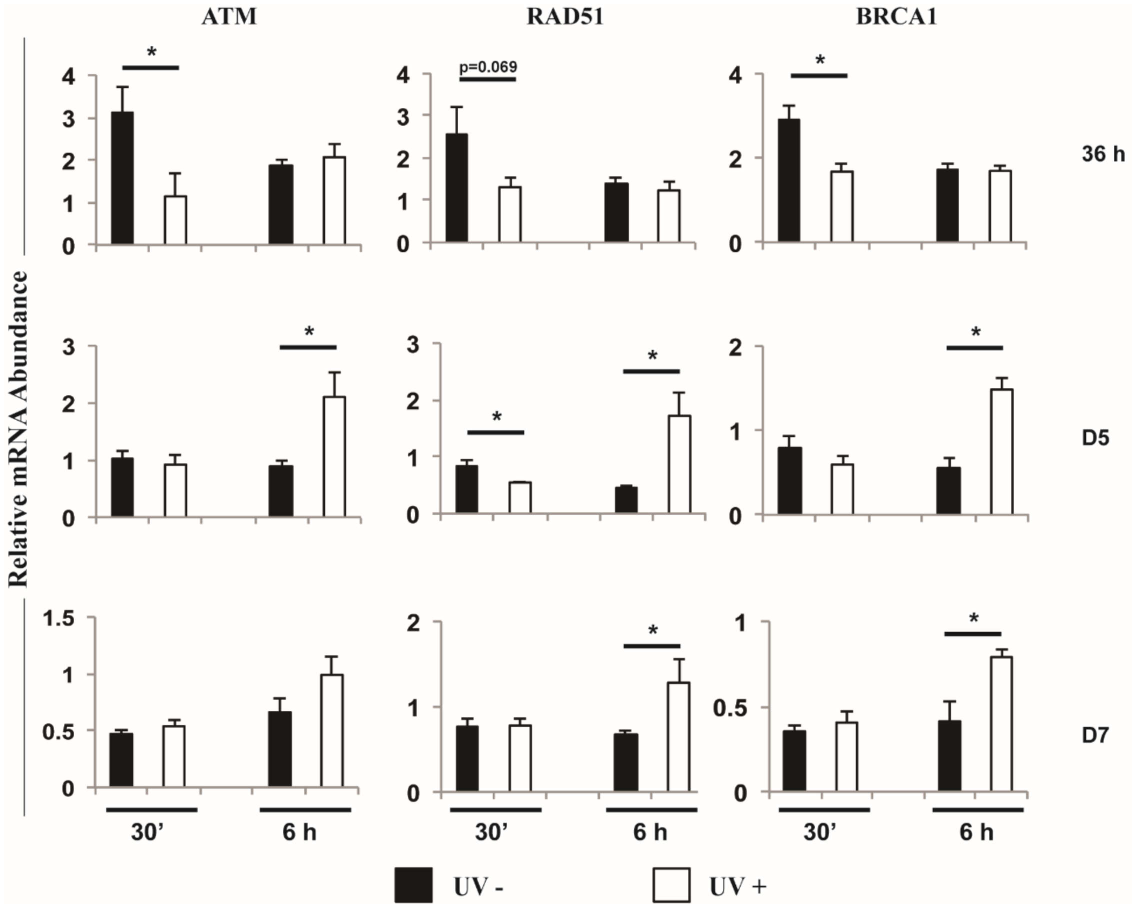

Fig. S2. Relative mRNA abundance of *ATM*, *RAD51* and *BRCA1* in embryos that were exposed (UV+) or not (UV-) to UV radiation for 10 seconds at 36 h, Day 5 or Day 7 post-activation. Samples from three replicates containing 10-15 embryos each were collected 30 min or 6 h after UV exposure and used for RNA extraction. (*) indicates statistical differences between UV- and UV+ groups within each developmental stage and time point ($P<0.05$).
